# Supplementary figures and images for: What are memories made of? A survey of neuroscientists on the structural basis of long-term memory
Source: PLoS One. 2025 Jun 24;20(6):e0326920. doi: 10.1371/journal.pone.0326920 (PMC12186944; doi:10.1371/journal.pone.0326920)

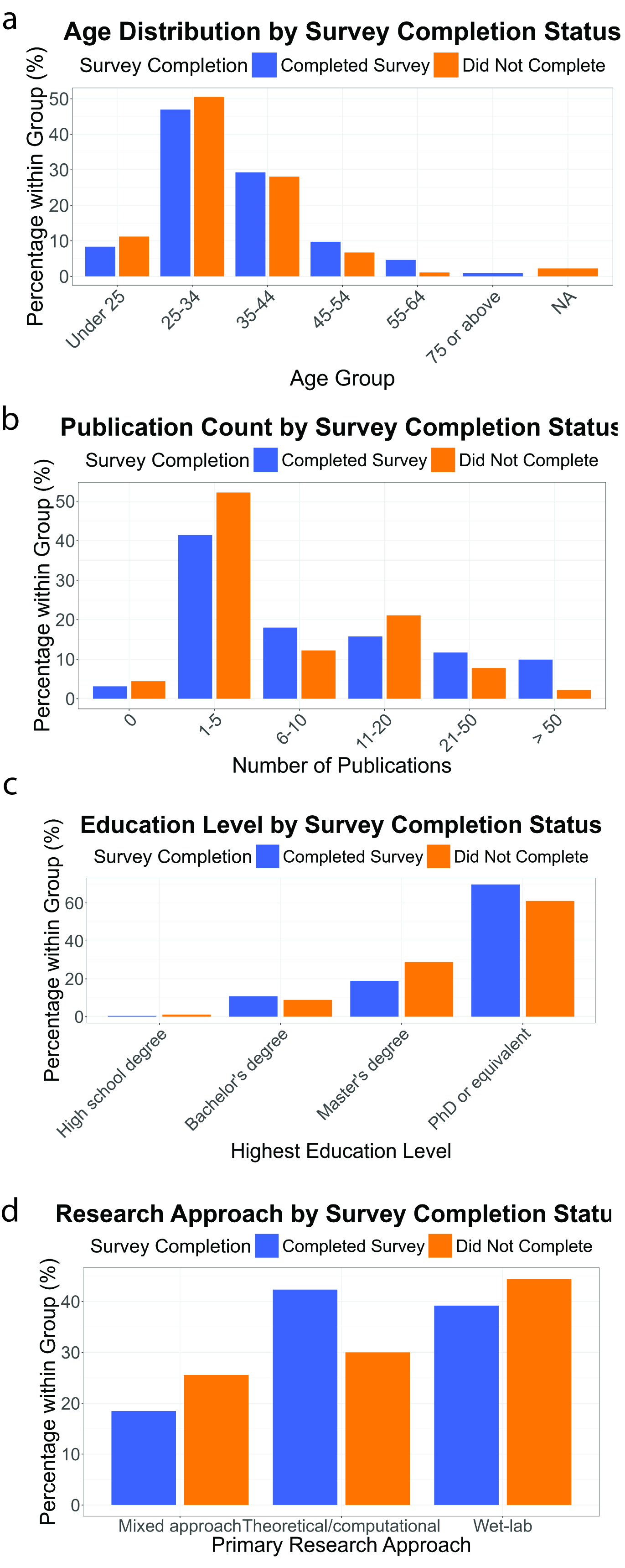

Supplement: S1 Fig — There were no differences between these groups based on: a) age (X: 10.384, df = 7, p = 0.17); b) publication count (X: 10.204, df = 5, p = 0.07); c) education level (X: 4.309, df = 3, p = 0.23); d) or research approach (X: 4.518, df = 2, p = 0.10). (TIF) [file pone.0326920.s001.tif]

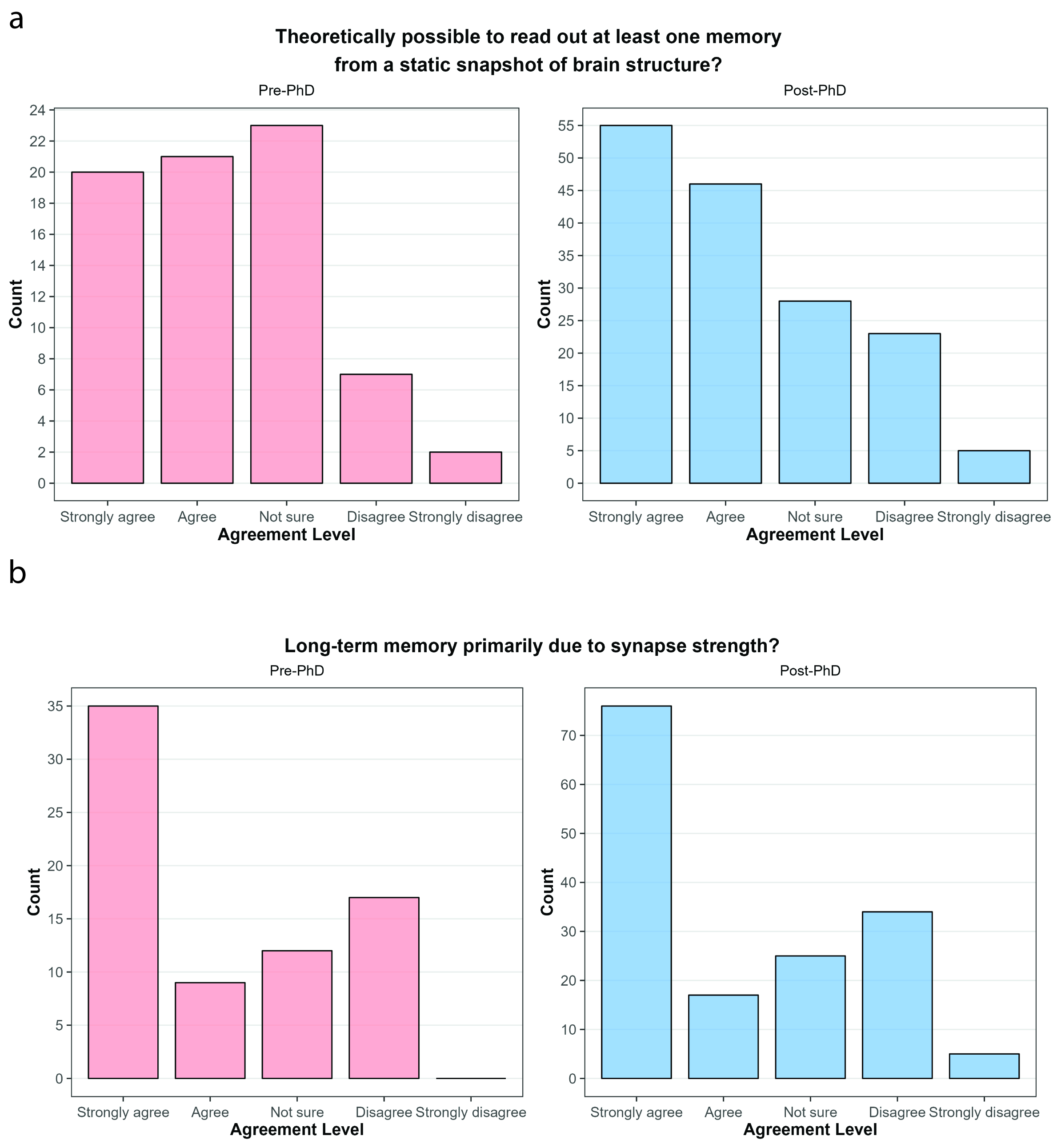

Supplement: S2 Fig — (TIF) [file pone.0326920.s002.tif]

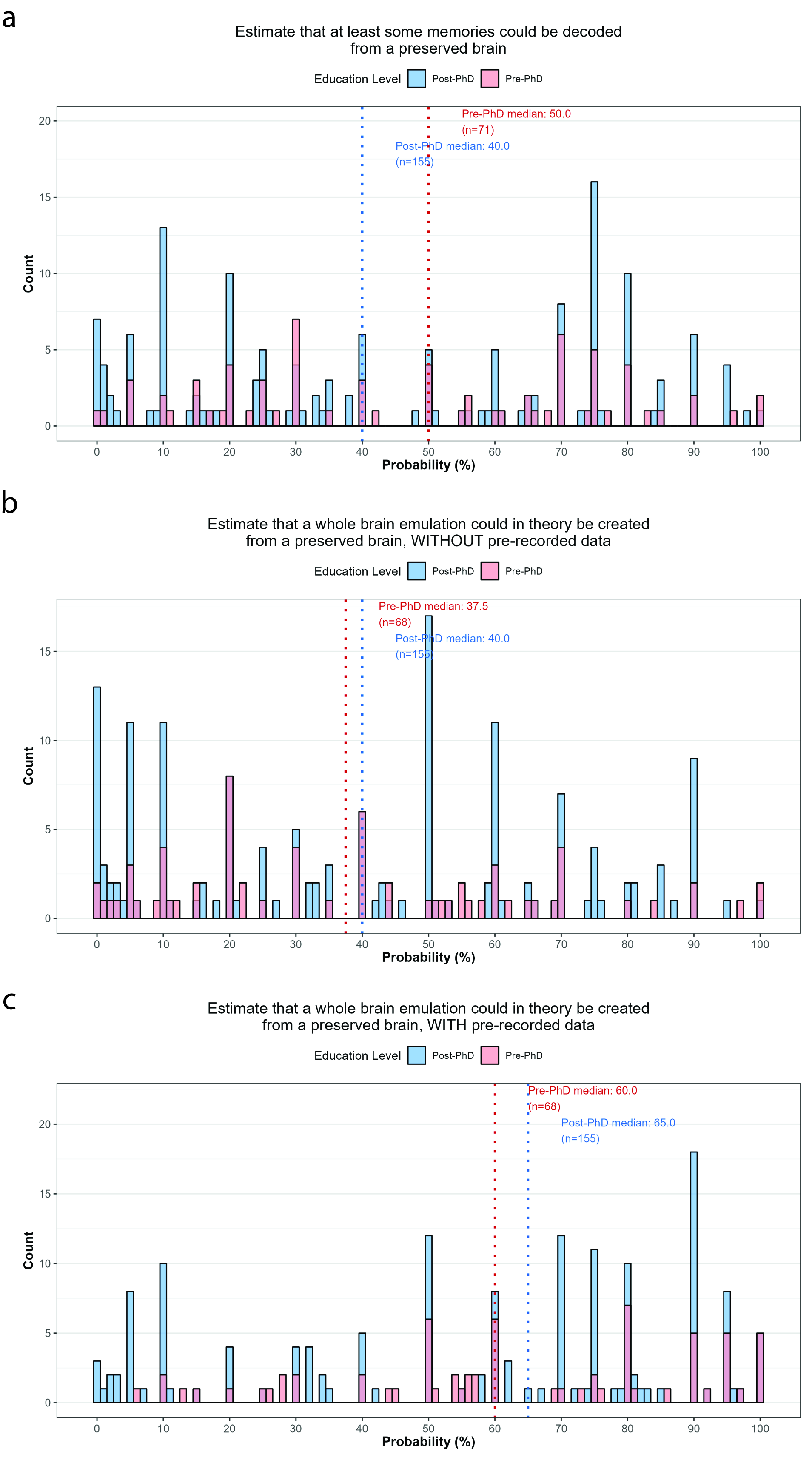

Supplement: S3 Fig — (TIF) [file pone.0326920.s003.tif]
