# Supplementary material for: What are memories made of? A survey of neuroscientists on the structural basis of long-term memory
Source: PLoS One. 2025 Jun 24;20(6):e0326920. doi: 10.1371/journal.pone.0326920 (PMC12186944; doi:10.1371/journal.pone.0326920)
Supplement: Attachment — Submitted filename: PLOS ONE Response to Reviewers - What are memories made of.pdf [file pone.0326920.s005.pdf]

Editor: In particular, the need to discuss intrinsic plasticity, as a potential cellular mechanism for memory formation is an important point.

We thank the reviewers for their helpful comments. Below, we provide detailed responses.

Reviewer #1: This manuscript aims to gather neuroscientists' opinions on the structural basis of memory storage and to provide a detailed analysis of the collected data. This study indicates that the main physical support of memory is synaptic plasticity.
The paper is very clear, well written and informative. I have only relatively minor points.

We thank the reviewer for their many kind comments!

Minor point:
1. Figure 1C, 5th raw: please correct Bimolecule by Biomolecule.

Corrected.
2. Page 18, 3rd line: changes and not chances

Corrected.

3. It is not clear in the manuscript what other mechanism could support the formation of memory. Are the authors thinking at intrinsic plasticity that is due to changes in voltage-gated ion channels and that is generally associated with synaptic changes (see for instance Titley et al., 2017 and Debanne et al., 2019)? A brief discussion of these aspects appears necessary.

We thank the reviewer for this comment. We agree about the need to be more explicit about what we mean by structural vs non-structural mechanisms, as well as to include intrinsic plasticity in the list of discussed memory-relevant neurophysiological mechanisms.

In paragraph 3 of the Introduction section, we have clarified structural vs non-structural properties by adding sentences stating:

"By 'structural,' we refer to relatively stable physical changes to neural components that persist independently of ongoing neural activity or metabolic processes, though these changes may initially require activity-dependent processes to be established. These structural changes exist on a spectrum from modifications of individual proteins and channel properties through to larger-scale alterations in neural connectivity."

We mean to differentiate these structural properties from things like persistent electrical activity or transient, short-lived, non-structural protein synthesis, which are known to sustain working or short-term memory.

Additionally, we have also added intrinsic plasticity into the list of potential structural mechanisms listed in paragraph 4. As the mechanisms mediating intrinsic plasticity involve features such as voltage-gated channel insertion into the membrane, post-translational

modification of these receptors, and alternate splicing, we have deemed these ‘structural’ (in opposition to the transient factors mentioned above).

Lastly, we have also added sentences to the third paragraph of the Discussion section stating that:

“ Nonetheless, when a mouse recalls the route through a maze, it is still unclear if this representation ultimately depends on the specific densities of receptors on particular dendritic spines in particular circuits, the coarse-grained connectivity strength of several populations of neurons, intrinsic plasticity manifested through persistent modifications of voltage-gated ion channels that alter neuronal excitability (8,9), or some combination of these or other neurophysiological features working in parallel.”

Reviewer #2: The article of Zeleznikow-Johnston, Kendziorra and McKenzie present the results of a survey they conducted on a sample of neuroscientists. The main goal of the study is to evaluate whether neuroscientists believe human brain can be stored, after fixation, in its native state ; and whether neuroscientists believe it is possible to access the content of a memory by looking at the fixed brain neuronal network.
The authors claims the goal of the survey is to establish whether the idea that memory is stored in patterns of patterns is a scientific consensus.
But the survey strangely goes into questions about emulation of a whole brain, reading memory content from a fixed brain.
One understands the reason behind a survey with such surprising questions when considering the conflict of interest statement. The authors are engaged in companies that offer the following service : store your brain after death, with the promise to read or do something from that preserved brain.

This reviewer is therefore in between two minds. On the one hand, the text of the article respects the rigor of science, and provides objective analysis, on the other hand the questions used in the survey do not assess the presented goal of evaluating a scientific consensus.

We thank the reviewer for this comment. In order to explain why we have asked these questions, which we agree might be confusing to a reader, we have added the following paragraph to the Discussion section:

“Although questions about ‘memory extraction from preserved brains’ and ‘whole brain emulation’ are admittedly strange and speculative, we believe they provide a practical framework for understanding memory's physical basis. These concepts create a direct link between theoretical views and their logical consequences. If memories are indeed encoded in stable structural elements like synaptic connectivity patterns - as 70.5% of our respondents indicated - then such memories should theoretically remain accessible in appropriately preserved neural tissue. Alternatively, if memories depend primarily on dynamic processes that cease at preservation, then extraction would be impossible. This approach is analogous to asking electrical engineers about data recovery from powered-down hard drives to understand their beliefs about how data is physically stored. We acknowledge, however, that while these questions provide valuable insights into neuroscientists' views on memory storage mechanisms,

they alone do not establish a scientific consensus on the precise physical basis of memory, as evidenced by the diversity of responses we received."

More precisely :

There is a problem with the sampling. The authors name « general neuroscientists » people who attended COSYNE congress in years 2022/23/24. Looking at speakers and titles of posters from the congress those three years, it appears the term « general neuroscientists » does not apply. It would apply when considering people who attended SFN or FENS.

COSYNE is clearly oriented towards network, computation, theoretical neuroscience. With some exception genetic and molecular levels are not represented, and experimental recordings are implemented at the level of the network, rather that single cell. There is also overrepresentation (as compared to what would be general neuroscientists) of computational and model neuroscience, which is a strong bias when question such as « could we emulate a brain in a computer » or « could we read information from the knowledge of the structure of a network » is given.
At minimum, the term « general neuroscientists » should be changed. Information on the orientation of COSYNE congress should appear in the article.

We thank the reviewer for this comment. We agree that the ideal version of this survey would involve surveying a substantial fraction of the participants at SFN or FENS, representing many thousands of neuroscientists from diverse subfields. While this is something we would like to perform in the future, unfortunately at present there is no publicly accessible database for contacting these participants. In contrast, COSYNE conference attendees are publicly listed in the conference abstract booklets, along with their email addresses, which made it possible to contact this entire cohort of participants in a systematic fashion.

Based on your comment, we have changed "general neuroscientists" to "COSYNE neuroscientists" throughout the manuscript, so as to be more precise about where this participant group originates from. We have also added an additional sentence to the methods where the participant cohorts are described, clarifying that 'COSYNE attendees are self-described as those interested in "the exchange of empirical and theoretical approaches to problems in systems neuroscience"'. We have also added a description in the limitations section of the discussion stating that "Third, we note that the COSYNE Neuroscientists comprise only one subsection of the neuroscientific community with a particular focus on computational and systems neuroscience, and that ideally this survey would be replicated with participants drawn from additional neuroscience subfields (such as attendees of the Society for Neuroscience or Federation of European Neuroscience Societies conferences). However, we note that the Engram Experts can serve as an internal control, as they were a non-COSYNE cohort of largely behavioural neuroscientists who have published studies on the neuroscience of engrams."

Asking "Given the scientific knowledge assumed in Point A existed, it would be theoretically possible to read out the information corresponding to at least one specific non-trivial memory

from a static snapshot of the structure (including biomolecules) of an organism's entire brain." to scientists attending SFN would give very different result.

On that question, one may also possibly expect that some people drop out of the survey when reading that question because they consider it is ill-defined (note that the question appears as the first question of the survey -not mentioning the 4 preeceding questions on the profile of the scientists such as age and level of education). This may represent a strong bias ?

We thank the reviewer for this comment. To partially address these concerns around potential bias, we assessed whether participants who dropped out after completing the initial demographics section differed in their demographic profile from those participants who completed the entire survey. We have added this additional analysis as a Supplementary Analysis to the manuscript, and added a description of this in the Methods section: "Participants who completed only the demographic section of the survey did not have a statistically significant difference in their demographic profile from those who completed the entire survey (Sup. Fig. 1). Specifically, completers vs non-completers did not differ significantly in terms of age (X: 10.384, df = 7, p = 0.17); b) publication count (X: 10.204, df = 5, p = 0.07); c) education level (X: 4.309, df = 3, p = 0.23); d) or research approach (X: 4.518, df = 2, p=0.10)."

Same for the question "The structural basis of long-term memories primarily consists of lasting changes in neuronal connectivity and ensembles of synaptic strengths, rather than in molecular or subcellular details.", people at SFN may not be 70 % to agree.

The categorization of the sample into either « Primarily computational or theoretical (e.g. dry-lab experiments » or « Primarily laboratory-based experiments (e.g., wet-lab experiments, neuroimaging, etc. » is not satisfactory. Neuroimaging is so far from wet-lab experiments, people doing neuroimaging are often from cognitive science.

We thank the reviewer for this comment. We have added this as a limitation of the study in the Discussion section: "Fourth, in terms of research background, those doing laboratory experiments can be distinguished into multiple types, including wet-lab experiments (patch clamping, animal behaviour, etc.) and neuroimaging techniques, which are substantially different. In future studies, it would be better to break down the participants into finer research background categories."

102 out of 312 « scientists » were at Bachelor or Master degree. Mixing students and researchers seems strange choice. How that population responded in comparison to the one having a PhD ? The time of PhD, and research time after obtaining the PhD, is important for one realizes better what is feasible and what science and technology can indeed bring. Please provide an analysis. And I do not see the level of eductaion in the correlation chart of Fig5.

We thank the reviewer for this comment. We have added this requested analysis to the Supplementary Analysis section, and noted this in the 'Influence of background and expertise' part of the Results section.

Supplementary Figure 2 displays how participants did not differ in their agreement as to whether it would be possible to read out memory from static brain structure based on whether they had obtained a PhD ($X = 6.375$, $df = 5$, $p = 0.27$), nor in their agreement as to whether long-term memory is primarily due to synapse strength based on whether they had obtained a PhD ($X=2.817$, $df = 5$, $p = 0.73$).

Supplementary Figure 3 displays how participant estimates that at least some memories could be decoded from a preserved brain did not differ for those with and without a PhD ($W = 5134.5$, $p = 0.42$). Additionally, participant probability estimates for whether a whole brain emulation could be created from a preserved brain without pre-recorded data did not differ for those with and without a PhD ($W = 5234.5$, $p = 0.94$), and the same was seen if the question was changed to include pre-recorded electrophysiological data ($W = 4724$, $p=0.22$)

Thank you for pointing out our error in having accidentally left education level out of the correlation matrix chart in Figure 5 - we have corrected this.

There are wrong and biased statements in the article.
1/ I am puzzled with the argument
« Long-term memories can be recalled even after periods of prolonged global neuronal depolarization and inactivity, as can occur in deep hypothermic circulatory arrest (2,3), or despite temporary protein synthesis inhibition (4). »

First, the ref 4, in its abstract, states : « Evidence from learning curves, examination of short-term retention, and posttraining drug injection indicate that initial acquisition is not dependent on such synthesis, but it appears that protein synthesis, during or shortly after training, is an essential step in the formation of long-term memory. » So it states the opposite for what the authors cites it.

We thank the reviewer for this comment. We agree that this sentence and citation is confusing, because we did not clearly define at the outset what we meant by the difference between 'memory formation' and 'memory recall.' To clarify this point, we have added text to the Introduction section just after this sentence to explain more clearly the point we were trying to make: "Additionally, while a temporary period of protein synthesis inhibition disrupts memory formation when administered during or shortly after training, it has been found to have no effect on memory recall when administered six or more days after training (4). This temporal distinction in the mechanisms between memory formation and recall suggests that long-term memory storage depends on stable structural changes rather than ongoing protein synthesis or dynamic neural activity, unlike working and short-term memory."

Second, why assuming from hypothermia it is evidence the electrophysiological and molecular level is not required ? If cooling, enzymatic activity will stop…and start again after temperature is back to physiological level. Electrophysiological activity as well.

If you wish to maintain your argument, please elaborate, and include and discuss the fact that

cooling make disappear dendritic spines, whihc reappear when warming after cooling
Kirov SA, Petrak LJ, Fiala JC, Harris KM. Dendritic spines disappear with chilling but proliferate excessively upon rewarming of mature hippocampus. Neuroscience. 2004;127(1):69-80. doi: 10.1016/j.neuroscience.2004.04.053. PMID: 15219670.

We thank the reviewer for this comment. We clarified our point about deep hypothermic circulatory arrest in the Introduction section: "Long-term memories can be recalled even after periods of prolonged global neuronal depolarization and inactivity, for example as can occur in deep hypothermic circulatory arrest (2,3). This suggests that ongoing global electrophysiological activity is not required for the retention of already formed long-term memories."

We also appreciate your reference to Kirov et al. (2004), which we agree is highly relevant to our manuscript. We believe that this reference is not only relevant to hypothermia, but also a good example of our broader point about structural resilience: despite temporary disruptions to fine structures like dendritic spines via cooling, the core information encoding long-term memories remains sufficiently preserved to allow recall upon rewarming. Therefore, we also added three sentences to the Introduction section regarding dendritic spine changes during cooling, at the end of the seventh paragraph: "For example, consider how dendritic spines on mature hippocampal neurons disappear when exposed to cold temperatures, yet reappear in the same locations upon rewarming (17). This finding shows that temporary disruptions to fine neural architecture can occur during certain perturbations (e.g. hypothermia) without necessarily destroying long-term memories (as demonstrated in humans who have undergone deep hypothermic circulatory arrest). For some, this may cast doubt upon any claim that dendritic spine positions are the exclusive substrate for long-term memory maintenance."

2/ The question "a brain successfully preserved with the aldehyde-stabilized cryopreservation method would retain sufficient information to theoretically decode at least some long-term memories?" may suffer from a bias
In the survey, the introduction before the question gives « If the procedure is successful, the combination of chemical fixation and cryopreservation is expected to allow stable, long-term storage of brains with minimal ultrastructural distortion. »
It seems to me the survey make a statement about the success of the method, and then ask the participant whether he or she believes in its success.

We thank the reviewer for this comment. We added a paragraph to the end of the Introduction section that clarifies what the goals of the study are and we believe addresses this concern: "For questions about the implications of static brain preservation for memory storage, we used aldehyde-stabilized cryopreservation (ASC) of a laboratory animal as a practical example of a preservation method that is thought to maintain ultrastructure with minimal distortions across the entire brain (24). Additionally, we asked participants to imagine it was performed under ideal conditions and was technically successful, deliberately discarding the fact that procedural variation or errors in the real world may prevent this ideal from being routinely realised in practice (25). Rather than focusing on these technical preservation challenges, which we acknowledge are immense, we deliberately asked participants to consider memory extraction under optimal preservation conditions to assess their beliefs about the structural basis of

memory storage itself. With this approach, our aim was to specifically target participants' views on whether static brain structures – i.e., non-dynamic physical aspects of the brain that persist independent of ongoing neural activity – may on their own contain sufficient information for memory retrieval, which is the central theoretical question underlying our study."

What if the introduction would have added the fact that learning and memory implies synaptic plasticity, which embbed morphological changes of synapses, and the fact that two fixation methods, aldehyde perfusion and cryo fixation, reveals different morphologies for the synapses ?
Korogod N, Petersen CC, Knott GW. Ultrastructural analysis of adult mouse neocortex comparing aldehyde perfusion with cryo fixation. Elife. 2015 Aug 11;4:e05793. doi: 10.7554/eLife.05793. PMID: 26259873; PMCID: PMC4530226.
Tamada H, Blanc J, Korogod N, Petersen CC, Knott GW. Ultrastructural comparison of dendritic spine morphology preserved with cryo and chemical fixation. Elife. 2020 Dec 4;9:e56384. doi: 10.7554/eLife.56384. PMID: 33274717; PMCID: PMC7748412.

We thank the reviewer for this comment. We have added a few sentences to the limitation paragraph of the Discussion section to cite both of these highly relevant articles. We discuss them in the context of our use of aldehyde-stabilized cryopreservation as an example of a preservation method that is thought to maintain ultrastructure, noting that some scientists might reasonably disagree with this characterization but that it does not necessarily invalidate the premise of our questions.

"Sixth, we note that our survey question assumes ASC would provide ideal-quality ultrastructural preservation, which may not be achievable routinely, especially outside laboratory settings, and depends on specific assumptions about ASC mechanisms. We note that some studies comparing preservation techniques have suggested that there are certain ultrastructural artifacts introduced by aldehyde fixation compared to cryofixed brain tissue (26,27). These findings are mixed, as more recent work has shown comparable measurements between techniques (28), and the preponderance of evidence suggests that the ultrastructural appearance achieved with both aldehyde fixation and cryofixation is largely similar (29)."

What if the introduction would remind to the participant the necessary time delay between postmortem brain extraction (the human subject will not stay next to the perfusion system, waiting for her or his death) and the perfusion ? It implies ischemia and cell death as well as synaptic loss.

We thank the reviewer for this comment. We agree that this is a highly relevant point that it is important to note. We have added a discussion of this as a part of discussing the limitations of our study in the Discussion section: "A valuable follow-up study would examine whether long-term memories might be preserved under more realistic conditions, such as when people donate their bodies to science. Unlike the ideal conditions assumed in our survey, these situations involve unavoidable brain deterioration due to the cessation of blood flow and associated tissue decomposition. Such circumstances typically include periods of ischemia prior to preservation, potentially resulting in cell death or synaptic loss over time"

In the survey, one could ask if, assuming preservation is ideal, memory content could be read form fiuxed brain ; but the question was « Given the scenario outlined above, and assuming that the preserved brain can be maintained indefinitely, what is your subjective probability estimate that a brain successfully preserved with the aldehyde-stabilized cryopreservation method would retain sufficient information to theoretically decode at least some long-term memories? »
Either participant would answer based on their knowledge, or based on the « scenario », but the scenario offered incomplet information.

We thank the reviewer for this comment. We agree that the scenario provided incomplete information and that this might be a factor in how respondents answered. We added this as a limitation in the Discussion section: "Fifth, although we attempted to provide background information through the preamble to the questions in our survey, this information was of course incomplete. Respondents have vastly different amounts and sources of knowledge relevant to this topic, which surely affected their responses to the questions."

I did not understood the correlation analysis. How to correlate « research approach » with « memory expertise » ? There are 2 research approaches (wet and dry experiment oriented) and 2 expertise (engramm expert and general neuroscientist), so 4 correlations can be calculated, rather than 1 ?

We thank the reviewer for this comment. We agree that the methods for this figure were not adequately explained. We have added a paragraph to the Methods section to explain this part further:

"To assess the relationships between participants' responses to different questions, we performed a rank correlation analysis across the relevant variables in our dataset that we could include. These included demographic variables (age, education level, PhD status, research approach, publication count), self-reported expertise ratings (memory expertise, preservation expertise, neural modeling expertise; each measured on a continuous scale from 1-5), and survey responses (theoretical possibility ratings, ASC probability estimates, emulation probability estimates, timeline predictions, etc.). In order to include them in this correlation analysis, ordinal variables were coded numerically. For example, research approach was coded as +1 (wet-lab), 0 (mixed), and -1 (dry-lab), while memory expertise was coded from 1-4 (not familiar to highly familiar). The cohort classification (engram expert vs. COSYNE neuroscientist) was analyzed separately in the subgroup analysis, not in this correlation matrix. After calculating correlation coefficients and corresponding p-values for all variable pairs, we applied a Benjamini-Hochberg correction to control for multiple comparisons with a false discovery rate of 0.05. Only the correlations that remained significant after this correction were shown in the figure."

Also, to answer your questions directly: the correlation coefficient represents the overall relationship between these variables across all participants. For instance, a positive correlation between research approach and self-assessed memory expertise would indicate that as values move from dry-lab (-1) toward wet-lab (+1), there tends to be an increase in self-assessed

memory expertise levels. And regarding your question about computing "4 correlations rather than 1": The suggestion to calculate four separate correlations (between each combination of research approach and expertise level) would be appropriate for a categorical analysis approach like contingency tables or chi-square tests. However, our correlation analysis treats these variables as continuous or ordinal scales rather than discrete categories. Our aim is to measure the overall monotonic relationship between two variables across their entire range of values, which we believe allows us to detect trends across the full spectrum of responses.

I found that to ask to each participant what he or she expect the other would answer is very interesting approach.
We thank the reviewer for this kind comment. Questions around meta-confidence and meta-performance are quite common in cognitive science, as they enable researchers to distinguish individual performance from the perception of their performance, or their beliefs from what they believe others believe.

Reading the raw data in the .csv table, some problems appear. It is minor but I wonder how the authors considered it for the analysis.

To what extent do you agree with the following statement: "Given the scientific knowledge assumed in Point A existed, it would be theoretically possible to read out the information corresponding to at least one specific non-trivial memory from a static snapshot of the structure (including biomolecules) of an organism's entire brain."

is followed by

If you think that the above statement would most likely not be possible, even in theory, what additional information beyond a static snapshot do you think would be required to read out a specific memory? (Select all that apply)_Not applicable, I do think this would most likely be possible in theory

a participant (line 6) selected : Strongly disagree
and then : Not applicable, I do think this would most likely be possible in theory

a participant (line 67) selected : Disagree
and then Not applicable, I do think this would most likely be possible in theory

a participant (line 205) selected : Agree
and add a general comment :
The question is ill-posed. In theory, with the right initial conditions, the locations and states of all the matter in the brain, and its coupling with the body is "all there is". But it isn't practical to reconstruct any of this. All known preservation and measurement processes would likely destroy the state irreparably.

Hence these 3 participant gave paradoxical answers

We thank the reviewer for this comment. Of the 312 participants who responded to our survey, a few participants occasionally provided responses that we find surprising, as per the examples that you listed above. An argument could be made for excluding such participants from the analysis, on the grounds that they seem to have potentially misread the question, or to be using an idiosyncratic interpretation of the possible response methods. However, post-hoc exclusion of participants introduces a substantial risk of bias and the potential for us as authors to manipulate the data to increase the chances of significant findings. Additionally, because we have a large number of respondents, removal of these three participants has negligible effect on any of the findings.

To address and acknowledge these concerns, including the ones listed below, we have added a paragraph in the limitations portion of the Discussion section stating:

"Last, we should flag that our analysis of the data is incomplete, as participants provided additional information through hundreds of free-text comments that this report has not examined. Informal exploration of these comments reveals nuanced perspectives that quantitative data alone cannot capture. For instance, participants who agreed that memories are primarily stored in synaptic connectivity sometimes qualified their responses with significant uncertainty, noting it could "go either way" or represented "wild speculation." Others highlighted definitional ambiguities, particularly regarding what constitutes "emulation" or how to interpret terms like "primarily." While we chose not to exclude potentially contradictory responses to avoid introducing selection bias, these comments suggest that many contradictions reflect sophisticated reasoning rather than misunderstanding. The qualitative insights underscore a central finding of our study: while there may be provisional agreement on broad principles of memory storage, substantial uncertainty remains about specific mechanisms and implications."

On wether the participants agree Poo's sentence ("The structural basis of long term memories primarily consists of lasting changes in neuronal connectivity and ensembles of synaptic strengths, rather than in molecular or subcellular details.")

line 130 stated
Agree
and add the comment :Again, wild speculation for now.

Line 169 stated
Agree
and add the comment : I could totally see it going either way, though. It may be that as a computational modeler, I *want* memory to be stored in connectivity, rather than molecular detail.

We thank the reviewer for this comment. The general point brought up is addressed in the paragraph we added about the free-text comments in the limitations portion of the Discussion section. For these comments in particular, we agree that they are interesting, while at the same

time think they are not necessarily paradoxical. These respondents might hold the position that they agree that long-term memories are encoded synaptically, but just with low confidence and an awareness of other possibilities.

« primarily » is a problem, two examples illustrate :

line 250 stated : Agree
and add the comment : I agree but I doubt that ongoing neural activity can be completely marginalized out such that only the connectivity matters

We thank the reviewer for this comment. The general point brought up is addressed in the paragraph we added about the free-text comments in the limitations portion of the Discussion section. For this comment in particular, this is why we also asked questions about emulation, because presumably this participant believes that decoding of at least some memories is plausible, but emulation providing a read out of *all* long-term memories is unlikely. Indeed, upon checking this participant's data, we found that they assign emulation without neural activity recordings a probability of success of 10%, whereas with neural activity recordings they increased it six-fold to 60%.

line 266 stated : Agree
and add the comment : I think this is quite likely given the current state of neuroscience knowledge, and it is the underlying tacit assumption of much current work in neuroscience. However, the brain frequently proves to be more complex than we thought, so it's difficult to exclude the possibility that more molecular or cell-autonomous mechanisms also play an important role.

When asked to estimate by which year a whole brain will be emulated, 4 participant sgave rather near years (such as 2025, 2035), while adding as comment :

272 not sure what degree of recreation you intend to achieve.
254 I am not sure what "emulation" means. Aren't there already efforts to do this and they consistently suffer from not having any reasonable measure of success.
143 this is a complicated question to answer: what is a "whole brain emulation"? Arguably this already exists (badly) for C. elegans, at least in terms of the electrophysiology.
97 Again it is very unclear to me how you would quantify a success in such a model of "emulation".

How such paradox in answers were treated in the analysis ?

We thank the reviewer for this comment. The general point brought up is addressed in the paragraph we added about the free-text comments in the limitations portion of the Discussion section. For these comments in particular, while we provided a substantial amount of context to specify what we meant by a whole brain emulation, in the context of a 20-minute survey, it is not possible to comprehensively address all questions participants may have. Notably though, we

did provide a definition, stating:

“In theory, a perfect whole brain emulation would replicate the internal causal structure of the original brain so closely that its behavioral responses to inputs and outputs would be indistinguishable from the biological version on which it was based.” and specifying “In contrast to *simulations* of neural activity that do not attempt to perfectly replicate the low-level internal parameters of a neuron (e.g. a single-point rate- firing neuron), the term *emulation* is used in this context because cells are modeled down to the lowest level of detail required to perfectly recreate their behavior (e.g. with a high-resolution compartment model, or even modeling each ion channel).”

Presumably, if we could outline in detail many different emulation scenarios with many different parameters, we would get slightly different participant timelines each time. However, as participants were asked merely to provide their best guess based on their own knowledge and the context they were provided with, their responses are still valuable when interpreted with these considerations kept in mind. Therefore, this point is also addressed by the other limitation which we added to the Discussion section based on your comments, as previously described: “Fifth, although we attempted to provide background information through the preamble to the questions in our survey, this information was of course incomplete. Respondents have vastly different amounts and sources of knowledge relevant to this topic, which surely affected their responses to the questions.”
